# Crumple: A Method for Complete Enumeration of All Possible Pseudoknot-Free RNA Secondary Structures

**DOI:** 10.1371/journal.pone.0052414

**Published:** 2012-12-27

**Authors:** Samuel Bleckley, Jonathan W. Stone, Susan J. Schroeder

**Affiliations:** Department of Chemistry and Biochemistry, Department of Microbiology and Plant Biology, University of Oklahoma, Norman, Oklahoma, United States of America; CSIR-Institute of Microbial Technology, India

## Abstract

The diverse landscape of RNA conformational space includes many canyons and crevices that are distant from the lowest minimum free energy valley and remain unexplored by traditional RNA structure prediction methods. A complete description of the entire RNA folding landscape can facilitate identification of biologically important conformations. The Crumple algorithm rapidly enumerates all possible non-pseudoknotted structures for an RNA sequence without consideration of thermodynamics while filtering the output with experimental data. The Crumple algorithm provides an alternative approach to traditional free energy minimization programs for RNA secondary structure prediction. A complete computation of all non-pseudoknotted secondary structures can reveal structures that would not be predicted by methods that sample the RNA folding landscape based on thermodynamic predictions. The free energy minimization approach is often successful but is limited by not considering RNA tertiary and protein interactions and the possibility that kinetics rather than thermodynamics determines the functional RNA fold. Efficient parallel computing and filters based on experimental data make practical the complete enumeration of all non-pseudoknotted structures. Efficient parallel computing for Crumple is implemented in a ring graph approach. Filters for experimental data include constraints from chemical probing of solvent accessibility, enzymatic cleavage of paired or unpaired nucleotides, phylogenetic covariation, and the minimum number and lengths of helices determined from crystallography or cryo-electron microscopy. The minimum number and length of helices has a significant effect on reducing conformational space. Pairing constraints reduce conformational space more than single nucleotide constraints. Examples with Alfalfa Mosaic Virus RNA and *Trypanosome brucei* guide RNA demonstrate the importance of evaluating all possible structures when pseduoknots, RNA-protein interactions, and metastable structures are important for biological function. Crumple software is freely available at http://adenosine.chem.ou.edu/software.html.

## Introduction

Amidst a flood of RNA sequence information and a tidal wave of new roles for small noncoding RNAs, successful exploration and navigation of the RNA world requires effective tools to evaluate structure and function from sequence. Many RNA structure prediction methods sample possible alternative structures in addition to computing the lowest free energy structure [1]–[5], but none rigorously explore all the conformational possibilities. The Crumple algorithm provides a method to compute completely and efficiently all possible non-pseudoknotted secondary structures for a given RNA sequence without consideration of thermodynamic parameters. Traditional free energy minimization methods do not consider stabilizing RNA tertiary interactions, RNA-protein interactions, or the possibility that kinetics rather than thermodynamics determines the functional structure. Crumpling an RNA sequence, like crumpling a piece of paper, is a fast and indiscriminate way of folding. Efficient parallel computing and effective experimental filters make the complete enumeration of all structures for an RNA sequence a reasonable approach. Experimental filters from chemical or enzymatic probing, phylogenetic analysis, crystallography, or cryoelectron microscopy can reduce the conformational space without overlooking structures that may be stabilized by tertiary and quaternary interactions.

The complete enumeration of the possible non-pseudoknotted pairings for a given RNA sequence is useful for defining the folding landscape, analysis of folding trajectories, and evaluation of experimental constraints. A set of possible folds from the output of the Wuchty [6] or Crumple algorithms is useful for mapping possible folding trajectories using programs such as BarMap [7]. Crumple can provide a larger set of structures that may allow additional insights into folding pathways. Crumple can also provide input for programs such as Sliding Windows and Assembly when the assumption of local cotranscriptional folding may apply [8], [9]. Complete enumeration of all possible solutions to the RNA folding problem allows a mathematically rigorous perspective. With a complete set of solutions for the folding problem, some hypotheses can be nullified. For example, the result that no possible solution exists for a given set of constraints can be computationally verified with mathematically complete methods. The effect of constraints on folding can also be evaluated in a more quantitative way with complete enumeration of possible RNA folds. Varying the application of constraints can provide new insights into the fundamental physical forces directing RNA folding.

Pipas and McMahon developed the first method to compute all possible folds for an RNA sequence [10]. In three steps, the method computed all possible helices of at least three pairs with at least a three-nucleotide turn, computed all possible combinations of those helices without allowing pseudoknot or non-nested interactions, and then ranked the resulting structures using free energy predictions. The Wuchty algorithm computes all possible structures within a given free energy window [6]. The algorithm does not limit the pairs in a helix, but rather limits the range of free energies of computed structures. In many cases, constraining the conformational space to a small thermodynamic range allows analysis of longer RNA sequences. The Helix Find&Combine program [8] computes all possible combinations of a defined helix length. Rather than use thermodynamics to constrain space, Helix Find&Combine uses minimum helix length with perfect pairing as a filter and allows non-nested pseudoknot interactions. In the case of satellite tobacco mosaic virus, this mathematically complete enumeration approach was used to nullify the hypothesis that the encapsidated RNA had 30 helices of 9 perfect pairs [8].

The Crumple algorithm is designed to compute efficiently all possible non-pseudoknotted pairings for an RNA sequence and allow flexible application of diverse experimental filters. For example, with efficient parallelization, complete enumeration of RNA sequences 50–60 nucleotides without any filters can be computed in 48 hours, the maximum time allotment on the Sooner supercomputer, an Intel Xeon E5405 2.0 GHz Linux MPI cluster. Filters such as covarying base pairs, chemical or enzymatic probing, no lonely pairs (single pairs without an adjacent stacking pair), the minimum number and length of helices, or thermodynamic stability can be incorporated into scoring functions. Comparisons of the effects of different filters demonstrate that pairing constraints reduce space more than unpaired single nucleotide constraints. Several examples with biological RNAs highlight cases when filters other than thermodynamic parameters identify important functional RNA conformations.

## Algorithm and Software Development and Methods

### Crumple Algorithm

It is natural to see the problem of producing every possible structure of a sequence as a recursive one; breaking a sequence into smaller and smaller parts does not change the nature of the problem. A subsequence can be treated like a complete shorter sequence. Combinations of base pairs create helices, and combinations of helices and unpaired regions create secondary structures. In order to reduce the complexity of the RNA folding problem for longer sequences, pseudoknot pairs, which involve pairs outside of the interval being considered in the recursion, are not considered.

**Figure 1:**
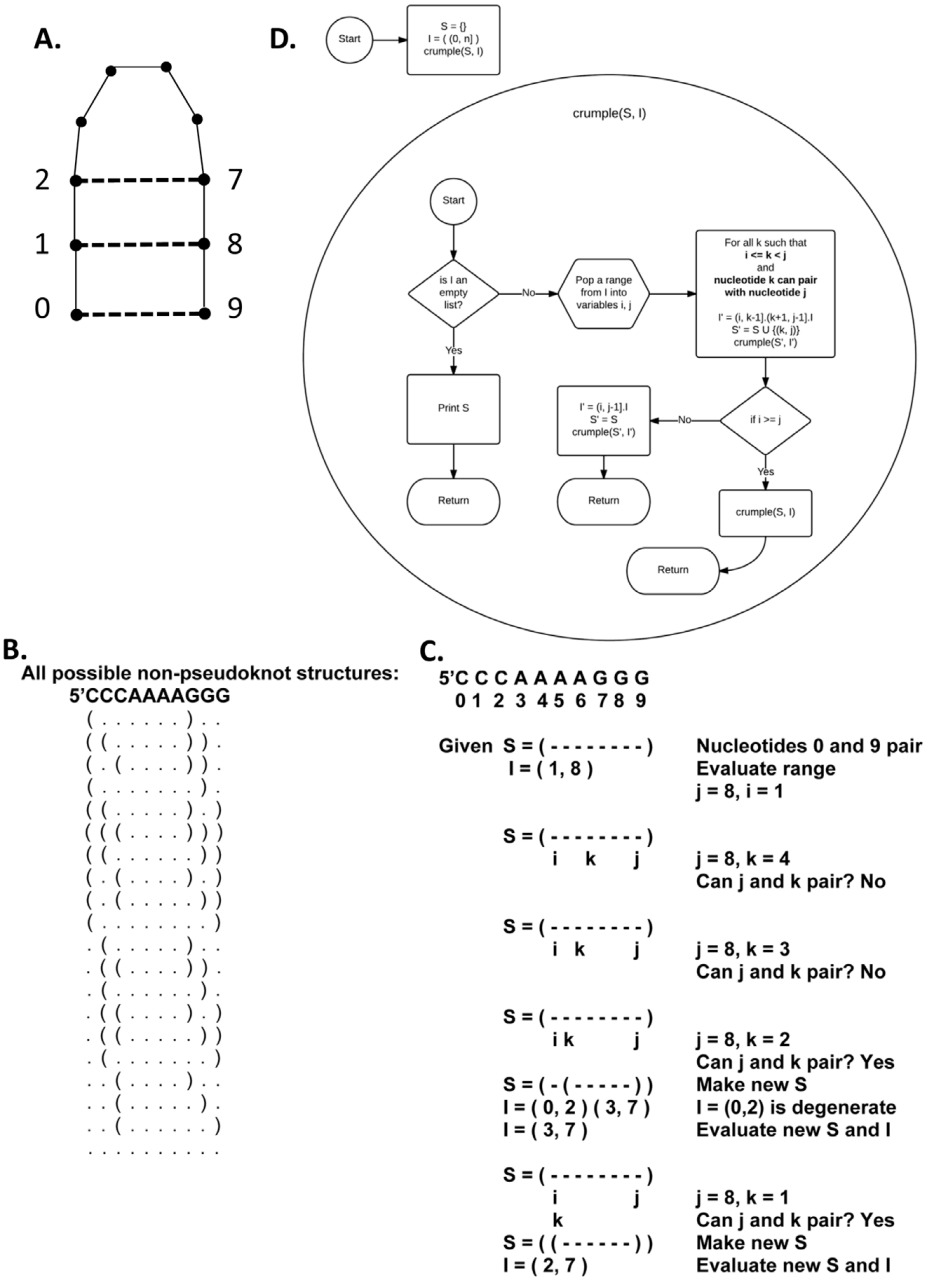
A. RNA Secondary Structure. Circles represent nucleotides. Solid lines indicate covalent phosphodiester bonds, and dashed lines indicate hydrogen-bonded base pairs. B. All possible non-pseudoknot pairs for the simple sequence 5′CCCAAAAGGG are listed below. Parentheses indicate pairs, and dots represent nonpaired nucleotides. C. An example of one step of the algorithm. Dashes indicate nucleotides that have not yet been examined. Dots indicate unpaired nucleotides. Parentheses indicate paired nucleotides. Nucleotide pairing follows Watson-Crick and GU pair rules. S is an RNA secondary structure or partial structure represented by a set of pairs (i,j). I is a stack of ranges (i,j]. Each range is a segment of the sequence that has yet to be examined. D. An empty list is defined as I = (). Adding an element X to a list is defined as I = X.I. Removing the most recently added element to the list, i.e. pop an element from the list, is defined as pop(I) =  = X, so that I =  = ().

In the Crumple algorithm, a secondary structure is defined to be a set of pairs of bases. The use of set theory notation facilitates the verification of completeness. The curly brackets define a set. Double curly brackets define a set of sets. Parentheses indicate a set where the interval boundaries are not included. Square brackets indicate a set where the boundary interval is included. The bold indicates the union of sets. The underlined question mark indicates the set to be determined. For example (Figure 1),

{(0, 9), (1, 8), (2, 7) … }.

A set of structures, then, is a set of sets of pairs of bases. Given a sequence s, the set of all possible structures formed in the subsequence between a base i and a base j is written in this way where i<j: 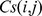. This set can be broken into two parts: the subset in which base i is paired, and the subset in which i remains unpaired i.e. 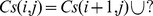, where ‘*?’* represents the subset in which base i pairs. In order to construct this subset an additional binary set operator is necessary and is equivalent to union mapped over cross product:
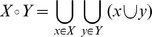



The set of all bases between i and j that can legally pair with j must also be defined:

pairs(i, j).

The set where i pairs, then is.
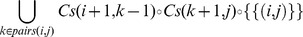



Which allows the definition of C_s_ to be completed:
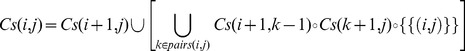



And so the full funnel of structures for a sequence s of length n is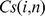.

However, while this is an extremely terse definition, a computational process based strictly on this recursion would rapidly exhaust its working memory: the size of the set C_s_ returns is exponentially related to the length of the subsequence, and the whole set of secondary structures must be held in working memory before it can be completed and saved to disk.

Exponential working memory is not fundamental to the problem, however. By a slight rearrangement, this definition can become an algorithm which is rapid but consumes memory linearly, completing structures one by one and saving them directly to disk.

Let I be a stack of ranges [i, j); each range is a segment of the sequence that has not yet been examined (the range includes the base at i but excludes the base at j). S is a partially formed secondary structure. S and I together will be referred to as a “state”. All the states that result from further crumpling of a state are “children”. The originating state is the “parent”. Then, (noting that the symbol+appends two lists):

crumple(I, S):

if |I| = 0:

 save S.

else:

 pop (i, j) from I.

 if i =  = j:

  crumple(I, S).

 for each possible canonical pairing (k, j) where i<k<j.

   crumple(([i, k−1), [k+1, j−1))+I, S **U** {(k, j)}).

 crumple(([i, j−1))+I, S).

crumple([1, |S|+1), 0).

Note that this pseudocode asks whether nucleotide j pairs with k, rather than i. This does not change the output, only the order in which it is created. This change was made to facilitate filtering structures that contain lonely pairs.

### Filters

Filters can be applied in several ways to help prune the enumeration of all possible structures and reduce both time and excessive output. Many filters simply check a partial structure to verify it contains no constraint violations, or check a potential pair to verify that it passes constraints. In implementation, even canonical Watson-Crick pairing rules are defined as a filter, preventing enumeration of structures containing illegal pairs.

A brief discussion of the lonely pair filter is an illustration of the way filters may be added to Crumple. Lonely pairs are isolated pairs surrounded by unpaired nucleotides. First, imagine that an additional step has been added to the very beginning of Crumple(I, S):

if lonely_pairs(S):

return.

Many filters can be added in this way and still separate folding and filtering functions.

lonely_pairs (S) could be implemented naively, examining each entire partial structure and looking for lonely pairs. This approach works, but is slow because it reexamines many perfectly acceptable pairs many times over. The simplicity of the Crumple implementation allows a more sophisticated approach to the lonely pair filter without creating unwieldy complexity in the code.

Each time a new pair is created, an interval is made inside that pair. The interval will be represented by the structure between [and ]:

….([………])….

(Note that there will be another interval created as well, along with all the pre-existing intervals, none of which are shown here.) At this moment, it is impossible to tell whether that pair will be lonely or not, and so there is no reason to examine it. In addition, the lonely-or-not condition of the pairs elsewhere in the sequence has not changed. In fact, the only moment when a pair can change its loneliness (lonely, stacked, or undetermined) is when an interval shrinks to length 0:

….([]………)….

At that moment, no more pairs can be made, and the pair immediately to the right of the shrunken interval moves from being indeterminate to (in this case) lonely.

Checking a single pair has a constant cost, while checking every pair becomes more costly as the length increases. Checking that single pair infrequently, *i.e.* only when the interval within reaches a length of 0, is more efficient.

### Parallelization

In implementation, the Crumple algorithm has been further transformed into a completely iterative process, and then parallelized, for the sake of speed.

Non-shared-memory parallelization presents two concerns: ensuring every process does a balanced amount of work, and minimizing the number of costly messages between processes. In the recursive version of Crumple, much of the potential work (i.e. the folding that has not yet been done) is implicit in the call stack. The only work that can easily be passed to another process is the work involved in completing the most recently created state. This represents a very small amount of the total potential work concealed in the call stack. That state represents the smallest unit of work extant in the process. Shuffling small amounts of work many times will result in good load-balancing, but it fails the second criterion: too many messages are sent, and the result does not scale well for many processes.

The greatest amount of work any single process has available is the parent state, from which emerges all the work to which the process has access. To use that knowledge, the recursion must be reified, making the whole set of partially completed states explicitly available (note: the number of states in this set is limited linearly with respect to the length of the sequence; therefore all necessary memory can be allocated initially, yielding a fortuitous increase in speed).

A 'continuation' state – one that has produced some, but not all, of its children – can be cleaved into two, according to two rules:

1. If there are still children to be examined from both the 'j pairs' case and the 'j remains unpaired' case of the current interval, produce one state that sires ONLY those children where base i pairs, and one state that produces only those children where i remains unpaired.
2. If there are only unexamined children from the 'j pairs' case (this can only result from a previous split due to rule 1), then divide the remaining pairing cases in half, and produce two states that each examine opposing halves.

While these two rules do not perfectly divide a process' remaining work in half, the approximation adequately satisfies the constraints of load balancing and minimal message passing. Table 1 shows efficient load-balancing in an example Crumple calculation with a guide RNA sequence.

**Table 1:** Work Distribution in Parallel Computations.

Number of Processes	Real Time (s)	Average Percent Work Per Process
4	42264	19.996±10.001
8	21118	11.107±3.956
16	10774	5.878±1.488
32	5630	3.206±0.570
64	3171	1.533±0.285
128	1926	0.771±0.180
256	999	0.384±0.073

The sequence in this example is a 48-nucleotide guide RNA. The computations were done in parallel on the Sooner supercomputer (Intel Xeon E5405 2.0 GHz Linux MPI cluster).

**Figure 2:**
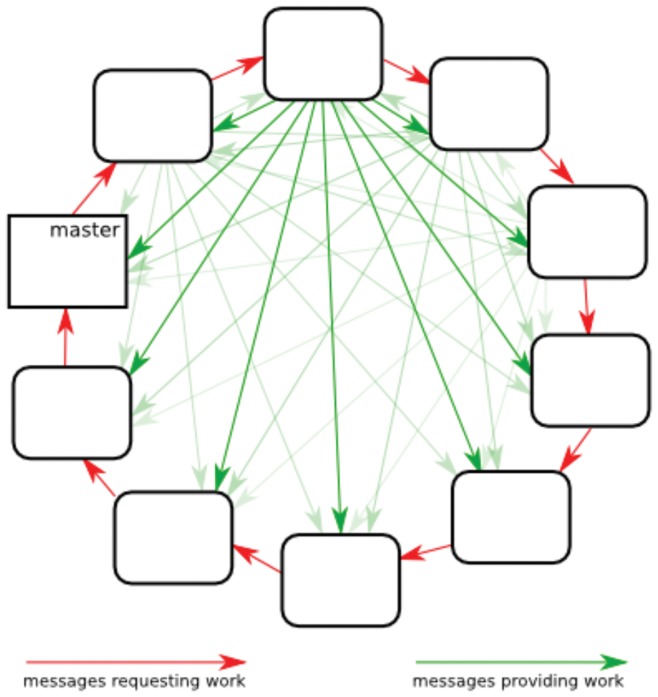
Ring Graph Parallelization Diagram for Crumple. Red Arrows indicate the direction of requests for work. Green arrows indicate the flow of distributed work. One node is arbitrarily selected as the first and master node.

In implementation, the individual parallel processes have been arranged in a ring graph (Figure 2). Each process communicates only with its neighbors, accepting requests-for-work from one side, and passing requests on to the other. Any process that receives a request for work must, if it has states available, cleave its root state, and send that work directly to the addressee. If no work is available, the work request must be sent along to the next node in the ring. Recognizing the halting-state is accomplished with a message passing algorithm reproduced directly from Dijkstra's work on token rings [11], [12].

### Additional Computational Methods

All software used in this work is freely available at http://adenosine.chem.ou.edu/software.html. The completeness of the Crumple algorithm was verified manually with a 14-nucleotide RNA sequence that has 119 possible structures (See List S1 and Supporting Information in reference [8]). The 14mer was specifically designed to include an example of each possible case for a chemically modified adenine. Concatenation of the 14mer to make 28mers and 42mers creates test cases that include multibranch loops. The Crumple algorithm has been applied to or tested on the following biological sequences: a section of the 5′ untranslated region of the HIV-1 RNA [8], satellite tobacco mosaic virus RNA [8], MS2 bacteriophage RNA [9], a section of the 3′untranslated region of Alfalfa Mosaic Virus RNA, 4 guide RNA from *Trypanosome brucei*, and a bacterial noncoding RNA MicA. In all cases, the minimum free energy structures predicted by rnafold, mfold, or RNAStructure were present in the Crumple output (Figure S1). The Crumple output was compared with the output from the Vienna implementation of the Wuchty algorithm [6], [13]. For longer sequences, the free energy of the structures generated by Crumple was computed and sorted and then compared to the output for the free energy window computed by the Wuchty algorithm. Crumple always computed at least the same structures generated by the Vienna implementation of the Wuchty algorithm and often more valid structures that were eliminated by default settings in the Vienna program. Tools to evaluate the differences between two RNA secondary structures and the free energy of an RNA structure, the functions rna_dist and rna_eval, respectively, were those available at http://www.tbi.univie.ac.at/RNA/in the Vienna websuite [13], [14]. The 2004 thermodynamic parameters from the Turner lab are used to evaluate the free energy of an RNA secondary structure [15]. Computations and programming were done on an AMD Athlon 64 X2 6400+3.2 GHz CPU with 4 GB RAM. Computations in parallel were done using OpenMPI 1.4 [16] on the Sooner supercomputer, which is available through the OU Supercomputing Center for Education and Research. The Sooner supercomputer trials were conducted on Intel Xeon E5405 2.0 GHz CPUs. The communication between nodes via Infiniband uses QLogic QLE7240 HCA cards and QLogic 9024 switches. The cluster runs Red Hat Enterprise Linux 5.0 x86_64 (kernel 2.6.18). The crumple program was compiled with gcc 4.1.2.

## Results and Discussion

**Figure 3:**
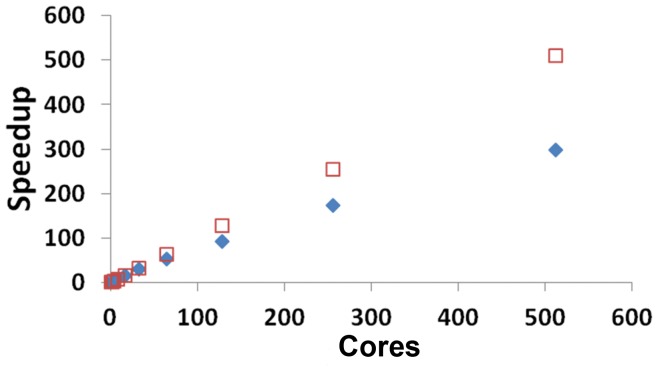
Parallelization Performance. Red squares are ideal speedup. Blue diamonds are the values measured using the gA48 sequence with no constraints on the Sooner supercomputer. Speed up is the ratio of real computation time in serial to real computation time in parallel. Note that one unit of work is assigned to each core, thus one node is equivalent to one core in this case.

Figure 3 shows an example of the speed up for a Crumple computation and demonstrates good performance for this parallelization scheme. Speedup is the ratio of times for the serial computation and the parallel computation. The speedup tests were performed with no experimental constraints on a 48-nucleotide guide RNA sequence from *Trypanosome brucei* that has a variable number of U nucleotides at the 5′ end [17]. The speedup is almost perfectly linear until 32 processes in this example. The point at which the graph of speedup shows nonlinearity will vary with the sequence and sequence length. Longer sequences with more work to be done will show more linear behavior for a greater number of nodes. The nonlinear behavior reflects too many requests for work for the given amount of work to be done. The actual time for the computation was 47.5 hours in serial and 30.9 minutes for 512 nodes on the Sooner supercomputer. In the best case scenario, Crumple completed computations for an unconstrained 60 nucleotide sequence with 1024 processes in the typical 48 h time allotted on the Sooner supercomputer. The speedup, time, and amount of work for a Crumple computation is sequence dependent. For a problem with exponential complexity and ideally linear speedup, doubling the number of processes approximately allows the consideration of a sequence one nucleotide longer in the same amount of time.

Table 2 summarizes Crumple computations for three biological RNAs of different lengths and with different amounts of experimental data defining the possible RNA conformations. The number of possible structures explodes exponentially with the sequence length [18]. For example in Table 2, the addition of 5 nucleotides increases the number of possible structures by 2 orders of magnitude. A sequence of 72 nucleotides, such as MicA, a small regulatory RNA in *Eschericia coli* [19], is still currently too long to completely enumerate all possible sequences without filters in two days time, which is the standard time period available on the Sooner supercomputer. Given the exponential growth of computing speed and memory predicted by Moore’s Law, however, the size of RNA folding problems that are reasonably computable will also continue to grow. The computing resources are not a fundamental limitation to the RNA folding problem. The ability to effectively filter or sort the large output in meaningful ways is the limiting condition. Constraining nucleotides to remain unpaired effectively shortens the sequence length in the MicA example. Thus filters with experimental data are the most effective approach to the task of enumerating all possible structures for a sequence. The computation is fast and efficient. Crumple calculations incorporating experimental constraints complete in less than a minute in serial on a standard PC computer.

**Table 2:** Examples of Crumple Computations for Biological RNAs.

RNA	Sequence Length(nucleotides)	Filters	Number of ComputedStructuresWithout Filters	Wall Time forComputation inSerial^a^	WallTime forComputation inParallel^b^	Number of ComputedStructures WithAll Filters	WallTime forComputation withAll Filters in Serial^a^
AMV4	39	3 single strand	50,781,504	40.89 s	2.75 s	91	0.01 s
RNA		2 chemical modification					
		10 paired					
		1 covarying pair					
		2 helices of at least 3 nts.					
		No lonely pairs					
Guide	44	6 single strand	5,370,612,993	1 h 17 m 5 s	46.2 s	53,009	7.08 s
RNA		11 chemical modification					
gND7-		2 helices of at least 3 nts.					
506		No lonely pairs					
MicA	72	27 single strand	-	-	>48 h	410,270,854	53 m 29 s
RNA		1 covarying pair					
		No lonely pairs					

a Wall time for computation in serial on a single AMD Athlon 64 X2 6400+ computer at 3.2 GHz with 4 GB RAM.

b Wall time for parallel computations with 256 processes on the Sooner supercomputer (Intel Xeon E5405 2.0 GHz Linux MPI cluster).

The Wuchty algorithm implemented in the Vienna RNA package [6], [14] and RNAStructure [20] is the only other algorithm that attempts to completely enumerate all possible pairings for an RNA sequence. The implementation of the no lonely pairs filter in the Vienna RNA package depends on the energy window and energy penalties rather than preventing the formation of lonely pairs (See Lists S1 and S2 for an example). The Vienna implementation of the Wuchty algorithm also limits the maximum pairing distance to 30 and the maximum energy window to 90 kcal/mol. The Vienna Wuchty program generates duplicate structures as a result of the different ways to compute dangling ends and multibranch loops. Because the Wuchty algorithm considers energetics before forming a pair, the algorithm also runs much more slowly than Crumple. In the cases of the AMV4 RNA and gRNA 7-506 RNA computed in serial in Table 2, the Vienna implementation of the Wuchty algorithm requires 2 min 33 s and 2 hours for the same computations on the same computer that Crumple requires only 41 s and 1 h 17 min hours, respectively. Thus, the Wuchty algorithm requires more time to compute fewer structures.

### Experimental Constraints Reduce the Conformational Space for the Minimal Protein Binding Site of Alfalfa Mosaic Virus RNA 4

**Figure 4:**
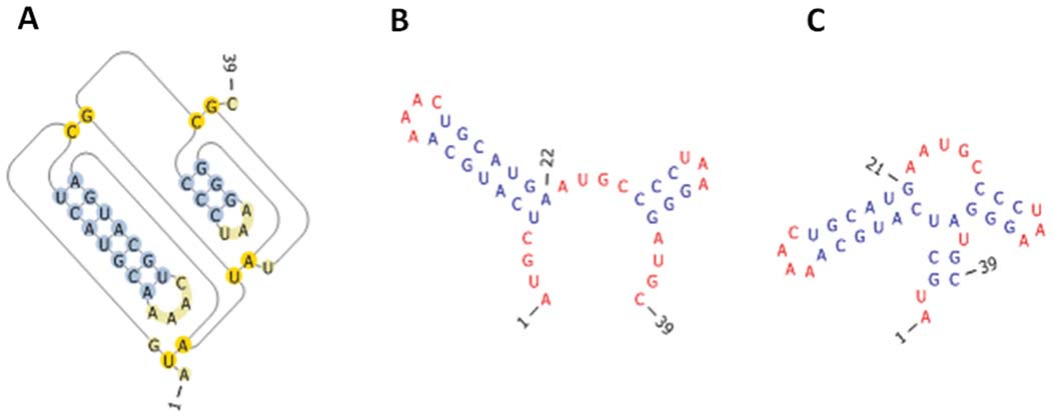
Alfalfa Mosaic Virus RNA 4 Protein Binding Site. A. Secondary structure with pseudoknots as seen in the crystal structure of the RNA-protein complex [21]. B. Secondary structure without pseudoknot interactions as determined by chemical and enzymatic probing of the RNA in isolation [22], [23]. C. Alternative AMV secondary structure containing a multibranch loop that is also consistent with the set of constraints listed in Table 3 legend. Secondary structures pictures were generated with RNA Pseudoviewer [47].

The 39-nucleotide segment in the 3′ untranslated region of Alfalfa Mosaic Virus (AMV) RNA 4 contains conserved AUGC repeats and the minimal coat protein binding site necessary for infection [21]–[23]. The RNA secondary structure has been probed chemically, enzymatically, and phylogenetically [22], [23], and cofolding of the RNA and protein creates additional RNA pseudoknot interactions [21]. More than one possible secondary structure satisfies all the chemical and enzymatic probing data in several regions of the AMV4 RNA [23]. The pattern of chemical and enzymatic probing changes in the presence or absence of proteins [22]. Thus, AMV RNA provides interesting RNA folding challenges because it includes repetitive sequences, pseudoknot tertiary interactions, and significant energetically stabilizing RNA-protein interactions. Traditional free energy minimization programs such as Vienna [14] or RNAStructure [15] predict the hairpin loops correctly but also predict additional base pairs that create bulges or multibranch loops in the lower stem helices. Free energy minimization disfavors single-stranded regions of RNA such as the nucleotides between hairpins in AMV RNA (Figure 4B). Prediction of tertiary interactions such as pseudoknots is computationally intensive [24]–[27]. No methods currently exist to directly predict RNA-protein interactions. The possibility of pseudoknot or RNA-protein interactions is one reason to include energetically suboptimal structures in predictions and explore a wide range of RNA conformational space. Using Crumple and experimental constraints identifies a set of structures that includes high energy structures with unpaired nucleotides that have the potential for stabilizing pseudoknot and RNA-protein interactions. Free energy minimization approaches have a tendency to maximize and thus overpredict base pairing (Figure S1). The complete set of possible structures enumerated by Crumple includes structures with less than maximal pairing and thus facilitates the evaluation of potential tertiary or quaternary pairing interactions.

Table 3 shows an example of how each type of experimental constraint contributes toward reducing the possible conformational space of the AMV RNA 4 protein binding sequence. The effect of each constraint is highly sequence dependent. Usually multiple constraints from chemical or enzymatic probing are used to define a secondary structure [28], but in this example, each constraint is introduced individually to demonstrate relative orders of impact for different types of constraints. Chemical probes such as dimethyl sulfate, kethoxal, or hydroxyl radicals modify solvent-accessible nucleotides. The chemical probing constraints allow chemical modification to occur in unpaired nucleotides, paired nucleotides at the end of a helix, or paired nucleotides adjacent to GU pairs [15]. In this example, the two chemically modified nucleotides U872 and A873 are consistent with all possible secondary structures and thus do not reduce the possible number of structures. Single strand constraints from S1 enzymatic digestion are more stringent and force the nucleotide to be unpaired. V1 enzymes cleave nucleotides in double stranded-RNA, and nucleotides hit by V1 are constrained to pair but the identity of the partner nucleotide is unconstrained. Covariation occurs when two nucleotides show compensatory changes that maintain Watson-Crick pairing in nucleotide sequence alignments. In a covarying pair, both nucleotides are constrained to pair with each other. A single covarying pair constrains not only those two nucleotides, but also constrains the nucleotides in between the covarying pair to pair with each other if pseudoknot interactions are not allowed. The minimum number and length of helices can be determined from cryoelectron microscopy or crystallography [8], [29]–[31]. In this example, this constraint involves at least 12 nucleotides and thus has a larger effect on reducing the possible conformational space. Constraining the minimum number of helices also eliminates many partially unfolded structures. Constraints to pair do more to reduce conformational space than single strand or chemical modification constraints in this example, which may be the results of eliminating many partially unfolded structures. All the constraints combined generate a set of 91 structures from over 50 million possible structures in this example. Representative secondary structures from the set are shown in Figure 4. All 91 structures share the same two hairpin loops and then the possible pairings in the stems and hairpin connections vary with a maximum number of 8 differently paired nucleotides, as calculated using the rna-distance tool in the Vienna package [14]. Crumple accurately produces the minimum free energy structures in the set of 91 structures. Computing the free energies of 91 structures and then sorting based on free energy is faster and more efficient than computing free energies at each step of the complete enumeration of all possible folds. The advantage of looking at the set of 91 structures is the identification of tertiary interactions among less stable structures or the identification of alternative structures.

**Table 3:** Experimental Constraints Reduce the Conformation Space for Minimal Protein Binding Site of Alfalfa Mosaic Virus RNA 4.

Constraints	Number of Computed Structures
None	50,781,504
2 Solvent accessible nucleotides	50,781,504
1 Single stranded nucleotide	43,117,777
3 Single stranded nucleotide	24,164,642
1 Paired nucleotide	11,436,079
No loney pairs	7,842,584
1 Covarying pair	3,476,410
2 Helices of at least 3 pairs	50,888
10 Paired nucleotides	39,596
All combined constraints	91

Table 3: The sequence for the AMV binding site with numbering according to (23) is 5′843AUGCUCAUGCAAAACUGCAUGAAUGCCCCUAAGGGAUGC881**.** The experimental constraints used are the following: nucleotides solvent accessible to chemical modification U872, A873; 1 nucleotide single stranded A 856; 3 nucleotides single stranded A856, A855, A854; 1 nucleotide double stranded A853; C869-G877 covary; 10 paired nucleotides A853, C852, G851, U850, A849, C848, G859, C860, A861, U862.

### Crumple Thoroughly Explores Alternative Structures for a Metastable Guide RNA

**Figure 5:**
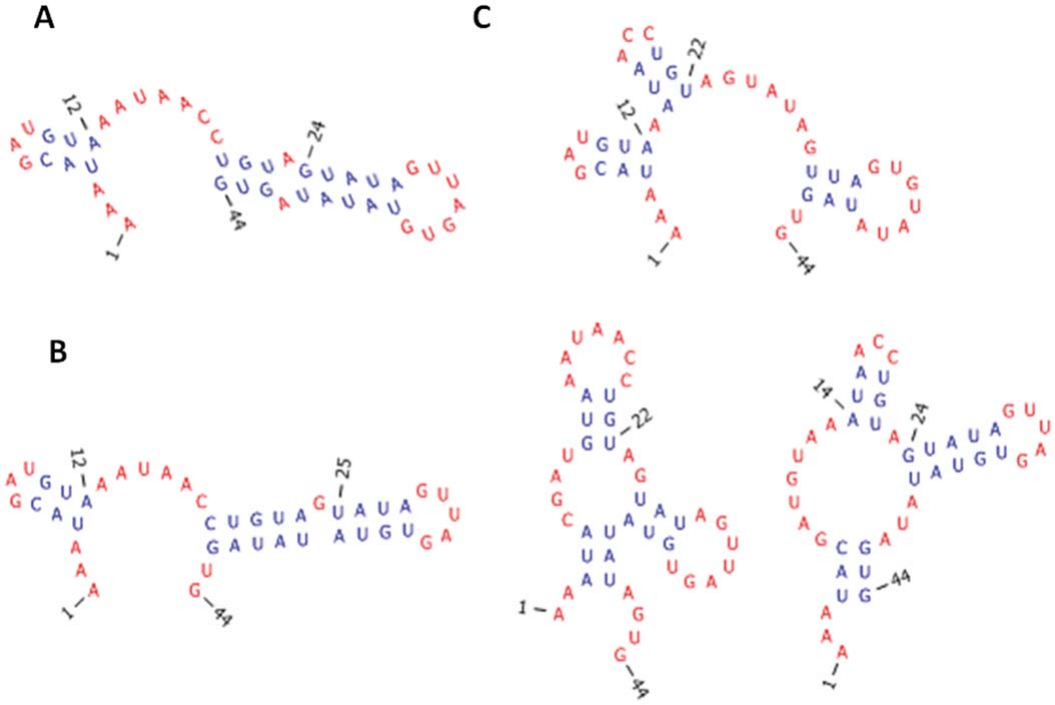
Guide RNA Secondary Structures. A. Secondary structure proposed from chemical and enzymatic probing of RNA *in vitro* [17]. In the protein-RNA crystal structure [36], only the first short hairpin is observed, and the second hairpin is unwound and only density for four nucleotides is observed. The structure shown in A has a predicted free energy greater than 0 kcal/mol [15]. B. Lowest energy secondary structure consistent with the given set of constraints. The predicted free energy is −1.6 kcal/mol. C. Alternative secondary structures that are consistent with the given set of constraints and that are not sampled by RNAStructure [48] or Sfold [49]. Secondary structures pictures were generated with RNA Pseudoviewer [47]. Experimental constraints include the following: chemically modified nucleotides A12, A13, A19, A24, A25, A27, G18, G21, G35, G40, G44 and single stranded nucleotides A12,A13,G18,U20,G40,G44.

Guide RNAs from *Trypanosoma brucei* pose unique RNA folding challenges. *Trypanosome* guide RNA sequences have a strong bias toward A and U and very few C nucleotides. Thus, the possible secondary structures contain mainly AU and GU pairs [17]. GU pairs show very idiosyncratic thermodynamic stabilities with non-nearest neighbor effects, and thus vary widely depending on the RNA sequence context [32]–[35]. Although proposed secondary structures were initially evaluated as energetically favorable (Figure 5A), the secondary structures would be unfavorable according to current thermodynamic prediction rules [15]. Metastable conformations play a role in the biological function of guide RNAs. Guide RNAs change conformation and partially unfold when bound by proteins; this protein-assisted RNA unfolding facilitates formation of the guide RNA-mRNA complex [36], [37]. Because this mechanism of RNA editing and U insertion into the mRNA sequence is unique to pathogenic *T. brucei*, guide RNAs are an attractive drug target.

Crumple predictions for the guide RNA segment used for the protein-RNA complex crystal structure show many possible structures that satisfy all the given constraints (Figure 5). Many of these structures contain sequence motifs for which thermodynamic parameters have not been measured and would be predicted to be unfavorable with the current thermodynamic prediction rules [15], [32], [35] that undergo continual evaluation and improvement. However, in the case of *T. brucei* guide RNA, the helix bound and stabilized by its protein partner is the helix predicted to have an unfavorable free energy rather than the helix predicted to have a favorable free energy. The lowest energy predicted structures are not consistent with the chemical and enzymatic probing data. Thus, currently predicted energetically unfavorable structures are worth consideration. The thermodynamic stabilities of GU pairs are an active area of research and are continually updated. Crumple can generate structures without reliance on these undetermined thermodynamic stabilities.

Note that double strand data from V1 enzyme was collected for this RNA also [17]. The hits from V1 enzyme, however, are not always consistent with the chemical probing data and apparently also strongly hit the tail of single-stranded, stacked uridines on the 3′ end of the guide RNAs. The strong propensity of V1 enzyme to hit both double stranded and single stranded, stacked nucleotides [38] in these conditions makes the data difficult to incorporate as a constraint for structure prediction. An alternative explanation of the apparent inconsistencies in the chemical and enzymatic data is that the guide RNA may exist in an ensemble of states. The complete enumeration of all possible structures with Crumple can facilitate the evaluation of an ensemble of RNA structures and its structural diversity.

### Applications of Crumple to Biological RNA Folding Problems

The advantages of using Crumple to generate all possible secondary structures include fast and efficient computation, a simple architecture that enables incorporation of experimental filters, and the ability to identify structures that may not be sampled by other methods. The disadvantages of this approach are that the number of structures grows exponentially with the number of nucleotides, many of the structures are very similar, and many of the structures have so few pairs as to be useless for practical biological problems. These disadvantages make the application of experimental filters essential to apply the approach to any biological problem. Thus, this method depends very much on the ability to define scoring functions and filters that effectively eliminate irrelevant structures and identify interesting structures. The simple architecture of Crumple facilitates the incorporation of diverse experimental constraints, and the design of the implementation explicitly considers future extensibility.

Programs such as BarMap [7] use the complete enumeration of structures, such as the output from Wuchty in the Vienna RNA Web Suite [14] or Crumple computations, to map possible kinetic folding trajectories. The ability to compute the complete set of possible structures with Crumple expands the potential applications of approaches such as Barmap to modeling RNA folding. The output of Crumple can also be used with programs such as Sliding Windows and Assembly [8], [9] to explore possible conformations of longer sequences, with local cotranscriptional folding. Thus, Crumple offers a useful alternative to traditional RNA folding methods.

The main utility of any RNA structure prediction or enumeration program is to generate hypotheses about structure and function in order to guide future experiments and further understanding of RNA molecules. From the practical point of view of an experimental biologist studying a new RNA, the following guidelines for the application of Crumple may be useful. Due to the large size of the output and the indiscriminate approach to RNA folding, the Crumple algorithm is better used after an initial traditional analysis of RNA secondary structure. A typical analysis might begin with folding the RNA sequence using minimum free energy methods, such as mfold [39], Unafold [40], Vienna [14], and RNAstructure [20] as a good starting point. Include as constraints all available experimental data, such as chemical or enzymatic probing, multiple sequences from phylogeny, covariation data, and base pairing from compensating mutations and functional analysis. If domains of longer RNA sequences have been identified or there are reasons to consider only local pairing, then fold individual domains or constrain the maximum pairing distance. Compare the minimum free energy structures from more than one prediction program. Analyze the base pairing probabilities calculated from the McCaskill algorithm [41], and observe the variation in the predicted suboptimal structures. If the predicted minimum free energy structures vary widely between different programs, then the prediction for this sequence may be very sensitive to the subtle differences in the implementation of the energy rules in different software programs. Low base pairing probabilities indicate multiple possible energetically stable structures for that region of the RNA. Keep in mind that the Zuker-Steigler algorithm generates representative suboptimal structureand will not combine two suboptimal structures formed by independent folding domains [5]. Crumple may provide additional insight into possible RNA structures if any of the following characteristics result from an initial traditional minimum free energy analysis:

1 the predicted minimum free energy structures differ significantly between different software programs;
2 the base pairing probabilities are lower than 50% for one or more regions of the RNA;
3 the predicted suboptimal structures vary significantly and have similar free energies;
4 pieces of different suboptimal predicted structures together would explain the data better than any single complete predicted suboptimal structure.

Crumple may also be useful if there are experimental data or hypotheses about function and mechanism that suggest the following:

5 RNA-protein interactions are significant;
6 pseudoknots exist; or
7 kinetics determines the functional structure.

If pseudoknots may exist, then consider using software programs that allow pseudoknots [24]–[27], [42]–[46]. Before using the Crumple tool, consider the length of the RNA and the available experimental data to filter the output. For example, crumpling one domain of an RNA structure with low predicted pairing probabilities may be more useful than crumpling the entire RNA sequence. The examples in Table 3 provide practical benchmarks to guide decisions about using Crumple. The Crumple tool provides a different view of the RNA folding landscape that can help an experimental biologist identify possible structures that may not be generated by traditional RNA structure prediction programs based on free energy minimization.

### Conclusions

Crumple provides a fast and efficient method to explore all possible conformations of an RNA sequence when the assumptions of free energy minimization may not hold true. Incorporating experimental constraints reduces the possible conformational space. Efficient parallel computing and filters from experimental data make complete enumeration of pseudoknot-free RNA structures a reasonable approach. This approach can facilitate the identification of secondary structures that enable stabilizing RNA tertiary and quaternary interactions and RNA-protein interactions.

## Supporting Information

Figure S1 **RNAStructure5.3 Minimum Free Energy Predictions for AMV, GDN-307, and MicA.**
(TIF)

List S1 **Output from Crumple and Wuchty computations for the sequence 5′GCUCUAAAAGAGAG.** Note: no filters and an energy window of 100,000 kcal/mol for the Wuchty computation
(DOC)

List S2 **Output from Crumple and Wuchty computations for the sequence 5′GCUCUAAAAGAGAG.** Note: no lonely pair filters for both computations and an energy window of 100,000 kcal/mol for the Wuchty computation.
(DOC)
