# Supplementary figures and images for: Crumple: A Method for Complete Enumeration of All Possible Pseudoknot-Free RNA Secondary Structures
Source: PLoS One. 2012 Dec 27;7(12):e52414. doi: 10.1371/journal.pone.0052414 (PMC3531468; doi:10.1371/journal.pone.0052414)

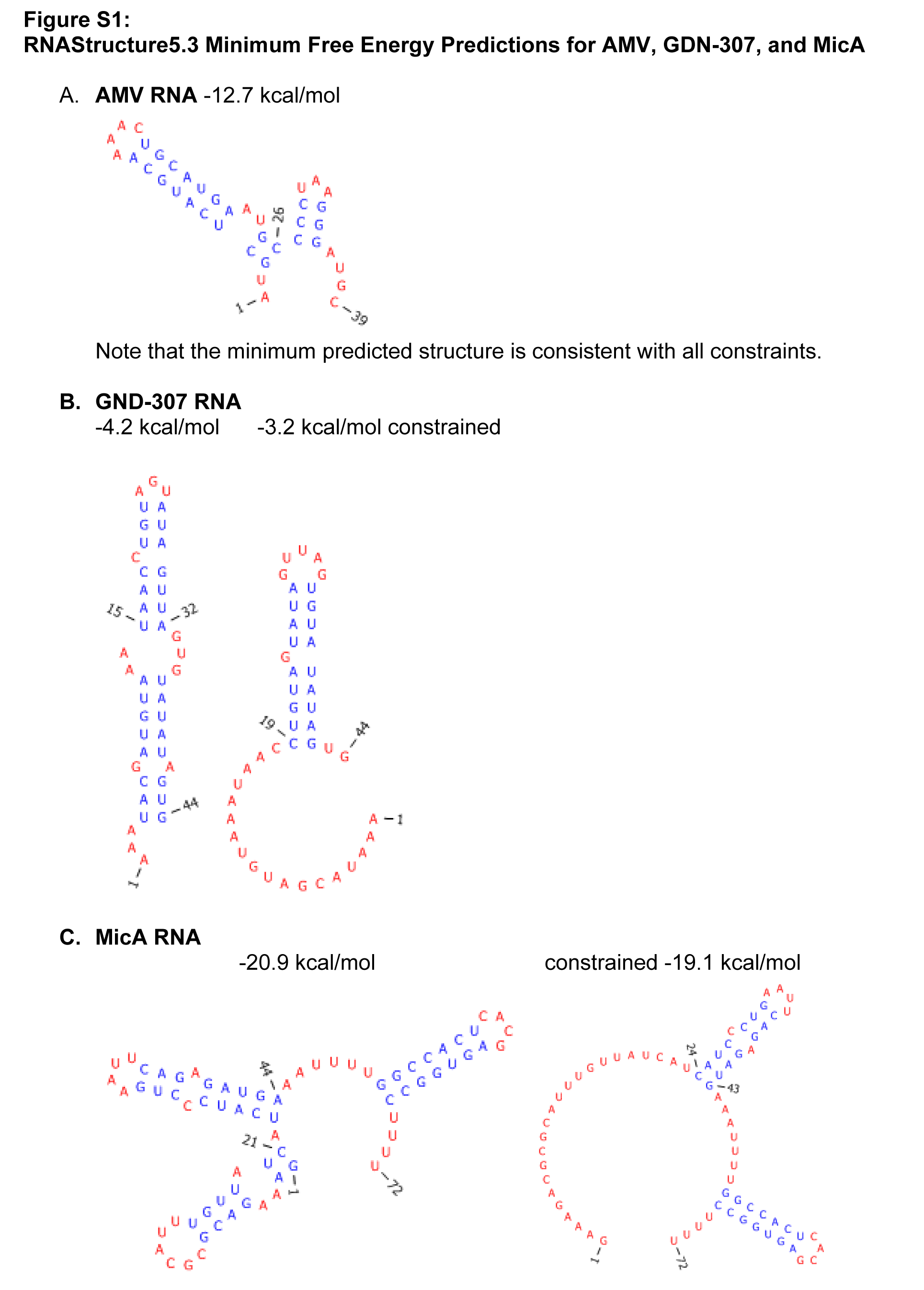

Supplement: Figure S1 — RNAStructure5.3 Minimum Free Energy Predictions for AMV, GDN-307, and MicA. (TIF) [file pone.0052414.s001.tif]
