# Supplementary material for: Crumple: A Method for Complete Enumeration of All Possible Pseudoknot-Free RNA Secondary Structures
Source: PLoS One. 2012 Dec 27;7(12):e52414. doi: 10.1371/journal.pone.0052414 (PMC3531468; doi:10.1371/journal.pone.0052414)
Supplement: List S1 — Output from Crumple and Wuchty computations for the sequence 5′GCUCUAAAAGAGAG. Note: no filters and an energy window of 100,000 kcal/mol for the Wuchty computation (DOC) [file pone.0052414.s002.doc]

Supporting Information for

Crumple: A Method for Complete Enumeration of All Possible Pseudoknot-Free RNA Secondary Structures

Samuel Bleckley, Jonathan Stone, and Susan J. Schroeder*

List S1: Output from Crumple and Wuchty computations for the sequence 5’ GCUCUAAAAGAGAG

Note: no filters and an energy window of 100,000 kcal/mol for the Wuchty computation

Crumple Wuchty

.............. ..............

....(........) ....(........)

> ....(........)

....(.......). ....(.......).

....(......).. ....(......)..

....(.....)... ....(.....)...

....(....).... ....(....)....

....(...)..... ....(...).....

...(.........) ...(.........)

> ...(.........)

> ...(.........)

...(.......).. ...(.......)..

...(.....).... ...(.....)....

...((.......)) ...((.......))

...((......).) ...((......).)

...((.....)..) ...((.....)..)

...((.....)).. ...((.....))..

...((....)...) ...((....)...)

...((....).).. ...((....).)..

...((...)....) ...((...)....)

> ...((...)....)

...((...)..).. ...((...)..)..

...((...)).... ...((...))....

..(..........) ..(..........)

> ..(..........)

> ..(..........)

> ..(..........)

> ..(.........).

..(.........). ..(.........).

> ..(.........).

> ..(........)..

..(........).. ..(........)..

..(.......)... ..(.......)...

..(......).... ..(......)....

..(.....)..... ..(.....).....

..(....)...... ..(....)......

..(...)....... ..(...).......

..(.(.......)) ..(.(.......))

..(.(......).) ..(.(......).)

..(.(......)). ..(.(......)).

..(.(.....)..) ..(.(.....)..)

..(.(.....).). ..(.(.....).).

..(.(.....)).. ..(.(.....))..

..(.(....)...) ..(.(....)...)

..(.(....)..). ..(.(....)..).

..(.(....).).. ..(.(....).)..

Crumple Wuchty

..(.(....))... ..(.(....))...

..(.(...)....) ..(.(...)....)

> ..(.(...)....)

..(.(...)...). ..(.(...)...).

..(.(...)..).. ..(.(...)..)..

..(.(...).)... ..(.(...).)...

..(.(...)).... ..(.(...))....

..((.......).) ..((.......).)

..((.......)). ..((.......)).

..((.....)...) ..((.....)...)

..((.....)..). ..((.....)..).

..((.....).).. ..((.....).)..

..((.....))... ..((.....))...

..(((.....)).) ..(((.....)).)

..(((.....))). ..(((.....))).

..(((....).).) ..(((....).).)

..(((....).)). ..(((....).)).

..(((...)..).) ..(((...)..).)

..(((...)..)). ..(((...)..)).

..(((...))...) ..(((...))...)

..(((...))..). ..(((...))..).

..(((...)).).. ..(((...)).)..

..(((...)))... ..(((...)))...

.(...........) .(...........)

> .(...........)

> .(...........)

> .(...........)

> .(...........)

> .(.........)..

> .(.........)..

.(.........).. .(.........)..

.(.......).... .(.......)....

.(..(.......)) .(..(.......))

.(..(......).) .(..(......).)

.(..(.....)..) .(..(.....)..)

.(..(.....)).. .(..(.....))..

.(..(....)...) .(..(....)...)

.(..(....).).. .(..(....).)..

.(..(...)....) .(..(...)....)

> .(..(...)....)

.(..(...)..).. .(..(...)..)..

.(..(...)).... .(..(...))....

.(.(.......).) .(.(.......).)

.(.(.....)...) .(.(.....)...)

.(.(.....).).. .(.(.....).)..

.(.((.....)).) .(.((.....)).)

.(.((....).).) .(.((....).).)

.(.((...)..).) .(.((...)..).)

.(.((...))...) .(.((...))...)

.(.((...)).).. .(.((...)).)..

.((.........)) .((.........))

Crumple Wuchty

> .((.........))

> .((.........))

> .((........).)

.((........).) .((........).)

.((.......)..) .((.......)..)

.((.......)).. .((.......))..

.((......)...) .((......)...)

.((......).).. .((......).)..

.((.....)....) .((.....)....)

> .((.....)....)

.((.....)..).. .((.....)..)..

.((.....)).... .((.....))....

.((....).....) .((....).....)

> .((....).....)

> .((....).....)

.((....)...).. .((....)...)..

.((....).).... .((....).)....

.((...)......) .((...)......)

> .((...)......)

> .((...)......)

> .((...)......)

> .((...)....)..

.((...)....).. .((...)....)..

.((...)..).... .((...)..)....

.((.(......))) .((.(......)))

.((.(.....).)) .((.(.....).))

.((.(.....)).) .((.(.....)).)

.((.(....)..)) .((.(....)..))

.((.(....).).) .((.(....).).)

.((.(....))..) .((.(....))..)

.((.(....))).. .((.(....)))..

.((.(...)...)) .((.(...)...))

.((.(...)..).) .((.(...)..).)

.((.(...).)..) .((.(...).)..)

.((.(...).)).. .((.(...).))..

.((.(...))...) .((.(...))...)

.((.(...)).).. .((.(...)).)..

.(((.......))) .(((.......)))

.(((.....)..)) .(((.....)..))

.(((.....).).) .(((.....).).)

.(((.....))..) .(((.....))..)

.(((.....))).. .(((.....)))..

.((((.....)))) .((((.....))))

.((((....).))) .((((....).)))

.((((...)..))) .((((...)..)))

.((((...))..)) .((((...))..))

.((((...)).).) .((((...)).).)

.((((...)))..) .((((...)))..)

.((((...)))).. .((((...))))..

(...).........
