# Supplementary material for: Crumple: A Method for Complete Enumeration of All Possible Pseudoknot-Free RNA Secondary Structures
Source: PLoS One. 2012 Dec 27;7(12):e52414. doi: 10.1371/journal.pone.0052414 (PMC3531468; doi:10.1371/journal.pone.0052414)
Supplement: List S2 — Output from Crumple and Wuchty computations for the sequence 5′GCUCUAAAAGAGAG. Note: no lonely pair filters for both computations and an energy window of 100,000 kcal/mol for the Wuchty computation. (DOC) [file pone.0052414.s003.doc]

Supporting Information for

Crumple: A Method for Complete Enumeration of All Possible Pseudoknot-Free RNA Secondary Structures

Samuel Bleckley, Jonathan Stone, and Susan J. Schroeder*

List S2: Output from Crumple and Wuchty computations for the sequence 5’ GCUCUAAAAGAGAG

Note: no lonely pair filters for both computations and an energy window of 100,000 kcal/mol for the Wuchty computation

Crumple Wuchty

.............. ..............

> ....(........)

> ....(........)

> ....(.......).

> ....(.....)...

> ....(...).....

...((.......)) ...((.......))

...((......).) <

...((.....)..) <

...((.....)).. ...((.....))..

...((....)...) <

...((....).).. <

...((...)....) <

...((...)..).. <

...((...)).... ...((...))....

> ..(..........)

> ..(..........)

> ..(..........)

> ..(..........)

> ..(.....).....

..(.(.......)) ..(.(.......))

..(.(......)). | ..(.(.....)..)

..(.(.....)).. | ..(.(...)....)

..(.(....))... | ..(.(...)....)

..(.(...)).... <

..((.......).) <

..((.......)). ..((.......)).

..((.....)...) <

..((.....)..). <

..((.....).).. <

..((.....))... ..((.....))...

..(((.....)).) ..(((.....)).)

..(((.....))). ..(((.....))).

..(((....).).) <

..(((....).)). <

..(((...)..).) <

..(((...)..)). ..(((...)..)).

..(((...))...) ..(((...))...)

..(((...))..). <

..(((...)).).. <

..(((...)))... ..(((...)))...

.(..(.......)) <

.(..(.....)).. <

.(..(...)).... <

.((.........)) .((.........))

.((........).) | .((.........))

Crumple Wuchty

.((.......)..) | .((.........))

.((.......)).. .((.......))..

.((......)...) <

.((......).).. <

.((.....)....) <

.((.....)..).. <

.((.....)).... .((.....))....

.((....).....) | .((.(.....).))

.((....)...).. | .((.(...)...))

.((....).).... | .((.(...).))..

.((...)......) <

.((...)....).. <

.((...)..).... <

.((.(......))) <

.((.(.....)).) <

.((.(....))..) <

.((.(....))).. <

.((.(...))...) <

.((.(...)).).. <

.(((.......))) .(((.......)))

.(((.....)..)) <

.(((.....).).) <

.(((.....))..) <

.(((.....))).. .(((.....)))..

.((((.....)))) .((((.....))))

.((((....).))) <

.((((...)..))) .((((...)..)))

.((((...))..)) .((((...))..))

.((((...)).).) <

.((((...)))..) <

.((((...)))).. .((((...)))).
